# Pain Relief in Cervical Dystonia with Botulinum Toxin Treatment

**DOI:** 10.3390/toxins7062321

**Published:** 2015-06-23

**Authors:** Carlos Henrique Ferreira Camargo, Lígia Cattai, Hélio Afonso Ghizoni Teive

**Affiliations:** 1 Neurology Service, Department of Medicine, Hospital Universitário, State University of Ponta Grossa, Ponta Grossa 84040-180, Brazil; E-Mail: clinicacattai@gmail.com; 2 Movement Disorders Unit, Neurology Service, Hospital de Clínicas, Federal University of Paraná, Curitiba 80060-900, Brazil; E-Mail: hagteive@mps.com.br

**Keywords:** cervical dystonia, pain, treatment, botulinum toxin

## Abstract

Dystonia is a neurological disorder characterized by intermittent or sustained muscle contractions that cause abnormal, usually repetitive, movements and postures. Dystonic movements can be tremulous and twisting and often follow a pattern. They are frequently associated with overflow muscle activation and may be triggered or worsened by voluntary action. Most voluntary muscles can be affected and, in the case of the neck muscles, the condition is referred to as cervical dystonia (CD), the most common form of dystonia. The high incidence of pain distinguishes CD from other focal dystonias and contributes significantly to patient disability and low quality of life. Different degrees of pain in the cervical region are reported by more than 60% of patients, and pain intensity is directly related to disease severity. Botulinum toxin (BoNT) is currently considered the treatment of choice for CD and can lead to an improvement in pain and dystonic symptoms in up to 90% of patients. The results for BoNT/A and BoNT/B are similar. The complex relationship between pain and dystonia has resulted in a large number of studies and more comprehensive assessments of dystonic patients. When planning the application of BoNT, pain should be a key factor in the choice of muscles and doses. In conclusion, BoNT is highly effective in controlling pain, and its analgesic effect is sustained for a long time in most CD patients.

## 1. Definition

The term torticollis was the first term used to describe cases of dystonia in the neck. In the 16th century, in his book Pantagruel, the renowned physician and playwright François Rabelais (1494–1553) described a giant with movement disorders of the neck and used the term *torti colli*, from the Latin: “...*afin qu’il ne fust torti colli*” [1]. In 1901, Joseph Destarac used the term *torticollis spasmodique* to describe dystonic neck and pelvic movements in a 17-year-old girl [2]. Destarac emphasized that these spasmodic movements occurred during activity and that they were relieved by rest and by various maneuvers [2]. The term dystonia was only coined in 1911, when Hermann Oppenheim described a muscle tonus disease and suggested the name *dystonia musculorum deformans* [3]. The definition of dystonia in 1984 was a seminal one but had several shortcomings. In view of these limitations, in 2013 the International Consensus Committee of the European Federation of Neurologic Societies proposed the following revised definition: *Dystonia is a movement disorder characterized by sustained or intermittent muscle contractions causing abnormal, often repetitive, movements, postures, or both. Dystonic movements are typically patterned, twisting, and may be tremulous. Dystonia is often initiated or worsened by voluntary action and associated with overflow muscle activation* [4]*.*

Most voluntary muscles can be affected, and in the case of the neck muscles, the condition is referred to as cervical dystonia (CD). CD is the most common form of focal dystonia [4,5,6,7,8,9]. It can also be part of generalized dystonia [7,8]. The involuntary contraction of neck muscles in CD leads to a wide variety of abnormal postures of the head. Torticollis is the name given to head rotation through the longitudinal axis toward the shoulder, while laterocollis refers to rotation of the head in the coronal plane, when the ear is moved toward the shoulder. Anterocollis and retrocollis refer to rotation of the head in the sagittal plane, the former taking the chin towards the chest, and the latter raising the chin and taking the occipital region toward the back [7,8,9].

## 2. Epidemiology

Dystonia is a relatively uncommon disease, and its prevalence varies according to the region studied. In Oslo, Norway, in a study carried out in 2003, the prevalences of primary focal dystonia and segmental dystonia were 25.4/10^5^ and 51.4/10^5^, respectively, in the population between 50 and 69 years of age [10]. The most prevalent form was CD. In Iceland, the prevalence of dystonia was 37.1/10^5^, and the predominant form was CD [11]. The most discrepant results were observed in Bruneck, in the Italian Alps, where the prevalence of dystonia was 732/10^5^ in people over 40 years of age [12]. A lower prevalence was observed among Asians than among Europeans and Americans (Nakashima *et al*, 1995). In Tottori, Japan, the prevalence of focal dystonias was lower (6.12/10^5^) than in Western countries; however, as in these countries, the predominant form was CD [13].

The incidence of CD found in Rochester was 1.2/10^5^. A comparison of the incidence of CD and other neurological diseases shows that CD is less common than Bell’s palsy (25.4/10^5^). However, it is more common than myasthenia gravis (0.3/10^5^) and has a prevalence comparable with those of amyotrophic lateral sclerosis (1.8/10^5^) and Guillain-Barré Syndrome (1.7/10^5^) [14].

## 3. Etiology

In the revisited classification of dystonias, the second axis addresses etiology, an area that is always changing and requires regular updating as new information becomes available. There are many forms of dystonia for which the etiology has yet to be elucidated. Two complementary characteristics can be used to do this: identifiable anatomical changes and inheritance pattern. While the former can be investigated by pathological examination or brain imaging, the latter requires genetic and metabolic tests. These two characteristics, anatomical changes and pattern of inheritance, should not be considered mutually exclusive means of etiological classification. For example, brain imaging can be helpful for identifying inherited and acquired dystonias, as MRI examination can reveal a perinatal lesion, indicating acquired dystonia [9].

Idiopathic dystonias are more common than acquired ones (Table 1) [15]. There are several causes for acquired dystonia, e.g., cervical trauma, cervical tumors, brain tumors, multiple sclerosis and heredodegenerative diseases such as Huntington’s disease and parkinsonism [16]. Tardive dystonia is the main form of secondary dystonia, with the cervical form being the most common [17,18].

**Table 1:** Etiology by distribution of cervical dystonia [15].

Etiology	Focal	Segmental	Generalized	Hemidystonia	Multifocal	Total
Idiopathic						
Sporadic	31	9	9	2	2	53
Familial	2	3				5
Acquired and Hereditary						
Neuroleptic Treatment	2	4	1			7
Perinatal anoxia	1	2	2	1		6
Craniocerebral trauma	5	1				6
Cervical trauma	3					3
Brain infarct	1	1				2
Meningitis				1		1
Behçet’s Syndrome				1		1
Wilson’s Disease			1			1
TOTAL	45	20	13	5	2	85

Over the past 20 years, several loci (from *DYT1* to *DYT25*) have been mapped in families with pure forms of dystonia, dystonia plus other movement disorders or sporadic cases. Until the recent description of the genes *CIZ1* (*DYT23*), *ANO-3* (*DYT24*) and *GNAL* (*DYT25*) associated with CD families, only *TOR1-A* (*DYT1*) and *THAP1* (*DYT6*) had been linked to isolated dystonia [18,19,20,21]. Craniocervical dystonia is one phenotype that can be associated with *DYT1* dystonia. It can be observed in the generalized form of the condition as well as in the segmental and focal forms [22,23]. The *DYT6* dystonia phenotype is highly variable even within a single family, ranging from the absence of signs and symptoms in unaffected carriers to generalized dystonia. It tends to remain in the focal or segmental form more often than *DYT1* [21,24,25].

## 4. Clinical Findings

Disability with functional impairment, pain and embarrassment leading to social withdrawal are frequent features of the disorder, and several studies have shown that CD can have an important impact on patient quality of life (QoL) [26,27,28,29].

### 4.1. Movement Disorder

Onset of CD is usually insidious although in 13% of patients symptoms may start suddenly [30]. Initially, patients typically complain of a “pulling” in the neck or involuntary twisting deviations of the head [7]. Often these nonspecific symptoms cause the physician to make an incorrect diagnosis of arthritis, cervical radiculopathy, psychiatric disorders or temporomandibular dysfunction. CD patients are often seen by several physicians before being referred to a movement disorders clinic for correct diagnosis [17].

The abnormal posture(s) in CD can be the result of any combination of the possible postures of the neck, which has many degrees of freedom because of the anatomy of the atlantoaxial joint and cervical spine, the large number of muscles and the complex origins and insertions of these muscles [31]. The most common presentation of CD is torticollis, followed by laterocollis, retrocollis and anterocollis [17,32,33]. Most studies show that a combination of deviations is more common, occurring in between 66% and 80% of cases [7,32] and that pure anterocollis is extremely rare [7]. Disproportionate anterocollis, characterized by severe neck flexion with minor thoracic or lumbar curvature, has been described in patients with Parkinson’s disease (PD) and has been suggested as a useful clue to the diagnosis of multiple system atrophy (MSA) [34,35].

The clinical behavior of patients with tardive dystonia has been observed to be different from that of idiopathic dystonia. In a study of the clinical characteristics of CD, Camargo *et al*. [15] found retrocollis in all their patients with tardive dystonia, and 85.71% of the patients had more than one type of dystonic movement, two characteristics that could be considered aggravating factors. The finding that retrocollis was predominant agrees with those of other authors who consider its presence to be highly suggestive of tardive dystonia [36]. Patients with tardive dystonia do not respond to treatment [15].

In some CD patients, sustained abnormal postures of the head may be associated with fast or slow intermittent movements and tremor. Twenty-four per cent of CD patients have neck spasms, 23% present with head jerks and 15% have both movements [32].

### 4.2. Pain

Pain is the main reason for patients to seek treatment for CD [31,32]. When asked how the symptoms of CD affected them, 66% of patients reported that they experienced a lot of pain [29]. In two studies, up to 90% of patients reported pain associated with CD [37,38]. Pain is a factor that greatly affects the QoL of these patients [39]. The intensity of the pain varies, but it is estimated that around 2/3 of patients require analgesics during the course of the disease [40]. A recent multicenter study showed that 88.9% (922/1037) of subjects reported pain associated with CD, 70.7% (733/1037 rated their pain as moderate or severe and 29.3% (304/1037) reported mild or no pain [38].

In over 85% of patients, the local pain associated with CD occurred in the neck, shoulder and back [32,41]. Rotation of the ipsilateral arm can induce pain in 50% of patients [41].

Pain in CD correlates with disease severity, but this relationship is complex. It is not clear whether the pain contributes directly to an increase in the severity of the condition or whether it is a consequence of increased severity of the condition [38]. The pain may result from strong muscle spasms (unlike in blepharospasm, for example, where the muscles involved are smaller and cause weaker spasms), the powerful force generated by the twisting movement of the neck, the time for which the muscles remain dystonic or possibly the high density of deep pain receptors in the neck muscles [32]. The pain perception threshold in CD patients is decreased; however, different discriminative aspects of pain in these patients can enable them to better establish their own threshold [42].

Although over 50% of CD patients complain of headache, the characteristics of this pain do not differ from those of headaches found in the general population. About 10% to 20% of CD patients have chronic daily headache, but only 1.3% of them meet the criteria of the International Headache Society for headache attributed to craniocervical dystonia [29,43,44]. Headache pain was reported in occipital (79.5%), cervical (72.7%), temporal (43.2%), frontal (36.4%) and retro-orbital (11.4%) areas and the vertex (25.0%) [29].

International headache society criteria for headache attributed to craniocervical dystonia [44]:

(A) Sensation of cramp, tension or pain in the neck, radiating to the back of the head or to the whole head and fulfilling criteria C and D;

(B) Abnormal movements or defective posture of neck or head due to muscular hyperactivity;

(C) Evidence that pain is attributed to muscular hyperactivity based on at least one of the following:

(1) Demonstration of clinical signs that implicate a source of pain in the hyperactive muscle (e.g., pain is precipitated or exacerbated by muscle contraction, movements, sustained posture or external pressure) and

(2) Simultaneous onset of pain and muscular hyperactivity;

(D) Pain resolves within three months after successful treatment of the causative disorder.

Many orthopedic and neurological complications causing cervical and radiating pain can be observed in CD patients. Among them, premature degeneration of the spine, spondylitis, vertebral subluxation, bone fusions, bone fractures, radiculopathy and myelopathy have been reported [45]. Although most authors establish differences between neurological and orthopedic symptoms, these are symptoms of an ongoing process. It is important to be alert for symptoms of radiculopathy or myelopathy so that treatment can be started or enhanced before the lesions in the nerve root or spinal cord become irreversible [45].

## 5. Pain Assessment

An ideal rating scale should be as short and simple as possible so that it can be implemented across multicenter clinical studies as well as in daily clinical practice. Not only functional disability but also the influence of the condition on activities of daily living and the psychosocial burden imposed on patient’s well-being need to be considered [46].

### 5.1. TWSTRS Pain Subscale

TWSTRS is the oldest tool for measuring severity, disability and pain in dystonia. It is valid, reliable and sensitive to changes in CD [31]. The TWSTRS pain subscale consists of a severity score for the patient’s usual, worst and best pain in the previous week, as well as a duration component and an assessment of the contribution of pain to disability [46].

### 5.2. Craniocervical Dystonia Questionnaire (CDQ-24)

The CDQ-24 is a disease-specific QoL instrument designed to investigate problems in daily-living skills related to CD and comprises 24 items within five domains related to stigma (questions 7, 8, 9, 10, 18 and 22); emotional well-being (questions 11, 12, 13, 14 and 15); pain (questions 4, 5 and 21); activities of daily living (questions 1, 2, 3, 6, 19 and 20) and social/family life (questions 16, 17, 23 and 24). Scores for each item range from 0 to 4 in increasing order of severity of impairment [47]. The validity of this instrument is questionable [31].

### 5.3. Patient Diary Items

Day-to-day capacities and activities, pain and duration of pain, and global assessment of pain [47].

### 5.4. Pain Numeric Rating Scale (PNRS)

The PNRS is a validated, single-item question on the current level of pain (range 0–10) with established cut-points of 0–3 for mild, 4–6 for moderate and 7–10 for severe. It is a widely used and recommended subject-reported measure. There are established cut-points, and the pain rating is independent of any other domain [48,49,50].

### 5.5. CD Impact Profile-58

This questionnaire consists of eight subscales (Head and Neck Symptoms, Pain and Discomfort, Upper Limb Activities, Walking, Sleep, Annoyance, Mood and Psychosocial Functioning), each ranging from 0–100. It can detect improvements in pain, activities of daily living and psychological and psychosocial functioning in patients with idiopathic CD following treatment with greater sensitivity than the gold-standard, TWSTRS. It is therefore recommended as the measurement tool of choice [31,32,33,34,35,36,37,38,39,40,41,42,43,44,45,46,47,48,49,50,51].

## 6. Treatment

BoNT, in particular BoNT/A and BoNT/B, has been successfully used to treat many neuronal disorders by taking advantage of its ability to interfere with a wide spectrum of physiological functions, ranging from a decrease in muscle contraction to pain alleviation [52]. Because of their high efficacy, tolerance, longevity and satisfactory safety profile, these botulinum toxins are now the most widely used therapeutic proteins [53].

BoNT is the treatment of choice for CD [52,53,54,55]. Its efficacy and safety in the management of CD are well established, and several studies have shown that treatment with this neurotoxin improves QoL in CD [47,56,57,58]. Approximately 50%–90% of patients experience improvements in dystonic symptoms and dystonia-related pain [59]. However, the advent of BoNT treatment did not eliminate the use of drug therapy in patients with CD because patients can develop antibodies to this toxin. Furthermore, some patients require a combination of treatments to achieve an acceptable reduction in pain and involuntary head movements [52,60].

When BoNT is injected into muscles, improvements in CD symptoms can usually be observed after one to 14 days. The peak effect typically occurs between two to six weeks after the injections, and the effect begins to wear off after 10–12 weeks. Some degree of muscle atrophy is observed after two weeks of treatment, and recovery of about 70%–80% of muscle mass occurs after three months [61].

The muscles to be injected vary according to the type of dystonia: for torticollis, the contralateral sternocleidomastoid and ipsilateral splenius; for laterocollis, the sternocleidomastoid, splenius, trapezius and scalene; for bilateral retrocollis, the splenius and trapezius; and for bilateral anterocollis, the sternocleidomastoid [59]. Injections can be administered in the ipsilateral or contralateral trapezius in cases of torticollis, and in the contralateral trapezius in cases of laterocollis [62].

Pain must be taken into consideration when determining the dose and the muscles to be injected [39]. It is useful to prioritize the importance of specific goals for the patient, such as reduction in pain, followed by improved ability to work [63]. The total dose of BoNT used in CD varies depending on the brand used. The recommended starting dose for treatment with Dysport^®^ is 500U, which results in significant benefits for most patients and minimal adverse effects [31,64]. Botox^®^ studies show that a dose of between 100 and 300 U is effective [65].

A number of studies have been conducted since 1986 to establish the efficacy of BoNT (Table 2) [15,33,64,66,67,68,69,70,71,72]. A prevalence of pain in the affected and adjacent regions and good pain response to local treatment with BoNT suggest a muscular mechanism for the genesis of pain [41]. It appears that BoNT does not stimulate central mechanisms of pain relief and that the improvement is probably caused by (**a**) a reduction in tone and volume, resulting in subsequent decompression of the nerve fibers, and (**b**) an increase in tissue perfusion, improving muscle metabolism by increasing oxygenation and eliminating sensitization [64]. However, some considerations about the initial interpretation that the pain originates from muscle disorders are in order. Firstly, one-third of patients do not report pain all the time, even with dystonic movements of similar severity and duration to those in patients suffering from pain; secondly, patients with pain do not relate it to the intensity of motor symptoms; and, lastly, the sensitivity of the cervical muscles varies little regardless of whether they act as agonists or antagonists or are not involved in dystonic movements, contradicting accepted explanations based on pain caused by excessive muscle work [41]. Furthermore, the improvement in pain without improvement in motor symptoms in patients undergoing bilateral stimulation of the globus pallidus and the presence of pain in areas far from the dystonia suggest a myofascial genesis and other central mechanisms of pain generation [41,73].

**Table 2:** Studies with BoNT for CD.

Study/Year	Patients	BoNT	Dose/Muscle (U)	Dose/Session (U)	Motor Response	Pain Relief
Tsui *et al*., 1986 [66]	19	Botox^®^	50	100	63%	87%
Gelb *et al*., 1989 [67]	20	Botox^®^	20–90	50–280	80%	50%
Jankovic *et al*., 1990 [68]	232	Botox^®^	20–200	100–300 average 209	70.7%	76.4%
Blackie and Lees, 1990 [69]	50	Dysport^®^	120–480	average 875	83%	77%
Jankovic *et al*., 1990 [70]	195	Botox^®^	25–100	average 209	90%	93%
Barbosa *et al*., 1995 [33]	19	Botox^®^	-	100–270	100%	100%
Poewe *et al*., 1998 [71]	75	Dysport^®^	75–300	300–1000	72%	16%–35%
Wissel *et al*., 2001 [64]	68	Dysport^®^	100–350	500	86%	42%
Camargo *et al*., 2008 [15]	85	Botox^®^		100–280	94.1%	84.4%

While there is proof of the efficacy and safety of BoNT and its superiority over anticholinergic drugs, doubts still remain about the types and doses of toxins that are most useful in the treatment of CD. Odergren *et al*. [74] compared 38 patients who received Botox^®^ and 35 who received Dysport^®^ using a Botox^®^/Dysport^®^ dose-conversion ratio of 1:3. The average doses of Dysport^®^ and Botox^®^ were 477 U (240–720 U) and 152 U (70–240 U), respectively. There were no clinical or statistical differences between the two groups, and the incidence of complications was similar. Ranoux *et al*. [75], in a study with 54 patients, also compared Botox^®^ and Dysport^®^ using a Botox^®^/Dysport^®^ dose-conversion-ratio of 1:3 and 1:4. All three treatments (Botox^®^, Dysport^®^ 1:3 and Dysport^®^1:4) were effective. There were no statistically significant differences in the results for the groups treated with Dysport^®^ 1:3 and with Dysport^®^ 1:4. However, patients treated with Dysport^®^ had better outcomes than those treated with Botox^®^ after one month of treatment. There were more adverse effects in patients receiving Dysport^®^ although this was not statistically significant. Marchetti *et al*. [76], with a different study design and a two-year patient follow-up, found a variation in dose-conversion ratio between BOTOX^®^ and Dysport^®^ of 2:1 to 11:1 with similar adverse effects in both preparations. Unlike other authors, Marchetti *et al*. stated that BoNT preparations cannot be accurately compared using a dose-conversion ratio and that clinical decisions should therefore be taken independently of dose-conversion factors, which are not accepted by consensus.

A comparison of BoNT/A and BoNT/B for CD was carried out in a multicenter controlled study with 139 patients. The results for pain relief and motor and disability improvements were similar for 74 patients receiving BoNT/A (maximum dose 250 U) and 65 receiving BoNT/B (maximum dose 10,000 U). There were, however, differences in the complications caused by these two types of BoNT, xerostomia and dysphagia being significantly more common in patients receiving BoNT/B [62]. BoNT/B is effective and safe for treating CD, has similar potency to BoNT/A and can be used in patients who are refractory to treatment with the latter [76,77,78,79,80].

A major cause of treatment failure with BoNT/A is toxin resistance. Long-term BoNT use may result in the production of neutralizing antibodies, which can impair clinical efficacy. A higher dose per session and frequent injections are associated with increased risk of developing immunoresistance [81]. Therefore, patients should be injected with the minimal dose necessary to provide a satisfactory result. Since injection frequency has also been associated with increased risk of immunoresistance, “booster shots” are generally discouraged and injection frequency should also be kept to a minimum depending on the particular circumstances [63,82]. Jankovic and Schwartz [83] demonstrated a 100% correlation between treatment failure and the presence of antibodies. These are more common in CD patients than in other dystonia patients, probably because of the higher doses of toxin required to improve CD. However, anti-BoNT antibodies are present in 9.2% of CD patients who are responsive to treatment and can be observed after cumulative doses with short intervals between applications. This difference between patients may be explained by genetic predisposition [84,85,86].

Despite the extensive benefits of BoNT for CD patients, treatment cost is a major factor limiting its use. A pharmacoeconomic study with CD patients showed that the average cost of CD treatment is US$ 97 ± 29 per month, rising to US$ 228 ± 30 a month after treatment with BoNT. The increase in cost refers only to the price of BoNT injections as spending on hospital care and diagnostic procedures decreases after treatment with this neurotoxin. Furthermore, the measured costs did not include the clinical improvement and the impact of pain relief, reduced social isolation and improved emotional behavior on QoL [87].

Physiotherapy can be combined with BoNT/A injections. There is evidence that a multimodal physiotherapy program consisting of active exercises, stretching and relaxation in addition to BoNT/A treatment induces beneficial effects on pain alleviation and disability in CD patients [88].

## 7. Final Considerations

BoNT/A is considered the treatment of choice for CD. It is a safe and effective form of treatment for motor symptoms and pain. When planning the application of BoNT, pain is an important factor that should be taken into consideration in the choice of muscles and doses. In addition to providing motor improvement and pain relief, BoNT/A improves patients’ QoL and social life, corroborating this choice of treatment for CD.
